# Effect of Continuous Positive Airway Pressure on Adiponectin in Patients with Obstructive Sleep Apnea: A Meta-Analysis

**DOI:** 10.1371/journal.pone.0136837

**Published:** 2015-09-14

**Authors:** Li-Da Chen, Jian-Nan Liu, Li Lin, Zhi Wu, Hao Li, Yu-Ming Ye, Qiao-Zhen Xu, Qi-Chang Lin

**Affiliations:** 1 Department of Respiratory Medicine, Zhangzhou Affiliated Hospital of Fujian Medical University, No 59, Shenglixi road, Xiangcheng district, Zhangzhou, Fujian province, People's Republic of China, 363000; 2 Fujian Provincial Sleep-disordered Breathing Clinic Center, Laboratory of Respiratory Disease of the Fujian Medical University, Department of Respiratory Medicine, the First Affiliated Hospital of Fujian Medical University, NO 20, Chazhong road, Taijiang district, Fuzhou, Fujian Province, People's Republic of China, 350005; Charité - Universitätsmedizin Berlin, GERMANY

## Abstract

**Objective:**

Obstructive sleep apnea (OSA) has been suggested to be associated with low levels of adiponectin. Continuous positive airway pressure (CPAP) is the gold standard treatment for OSA; however, previous studies assessing the effect of CPAP on adiponectin in patients with OSA yielded conflicting results. The present meta-analysis was performed to determine whether CPAP therapy could increase adiponectin levels.

**Methods:**

Two reviewers independently searched PubMed, Cochrane library, Embase and Web of Science before February 2015. Information on characteristics of subjects, study design and pre- and post-CPAP treatment of serum adiponectin was extracted for analysis. Standardized mean difference (SMD) was used to analyze the summary estimates for CPAP therapy.

**Results:**

Eleven studies involving 240 patients were included in this meta-analysis, including ten observational studies and one randomized controlled study. The meta-analysis showed that there was no change of adiponectin levels before and after CPAP treatment in OSA patients (SMD = 0.059, 95% confidence interval (CI) = −0.250 to 0.368, z = 0.37, p = 0.710). Subgroup analyses indicated that the results were not affected by age, baseline body mass index, severity of OSA, CPAP therapy duration, sample size and racial differences.

**Conclusions:**

This meta-analysis suggested that CPAP therapy has no impact on adiponectin in OSA patients, without significant changes in body weight. Further large-scale, well-designed long-term interventional investigations are needed to clarify this issue.

## Introduction

Obstructive sleep apnea (OSA) is a common medical condition characterized by recurrent and intermittent episodes of hypoxia during sleep due to the collapse of upper airways, resulting in fragmentation of sleep and excessive daytime somnolence[1]. OSA affects over 4% of the general population and 35–45% of obese individuals[2, 3]. A growing body of evidence supports the relationships of OSA with increased cardiovascular morbidity and mortality, and OSA has been considered to be an independent risk factor for cardiovascular diseases[4, 5].

There is an increasing recognition that adipose tissue is an endocrine organ secreting a number of biologically active adipokines such as leptin, adiponectin, and tumor necrosis factor-α. Among various adipocyte-derived adipokines, adiponectin has attracted considerable attention due to its role in cardiovascular disorders. It has been suggested that reduced serum adiponectin levels could partly explain increased cardiovascular disease in OSA patients. Previous studies showed that OSA was associated with significant decrease in serum adiponectin levels[6–8].

Continuous positive airway pressure (CPAP) is the most widely accepted treatment for OSA. Evidence indicated that treatment with CPAP can significantly reduce cardiovascular morbidity and mortality related to OSA[9]. However, whether serum adiponectin levels may be ameliorated or not by CPAP is unclear. The aim of the present meta-analysis was to quantitatively evaluate the impact of CPAP on adiponectin levels in OSA patients.

## Methods

### Search strategy

We comprehensively searched for English articles included in PubMed, Web of Science, Cochrane Library, and Embase up to February, 16^th^, 2015. Searches combined free-text and MeSH terms, and the combination of following search terms were used: “continuous positive airway pressure or CPAP”, “sleep apnea or sleep apnoea”, “adiponectin”. The reference lists of the included studies were also hand-searched. Two researchers independently identified the eligible studies. Conflicting decisions were resolved through a consensus with a third researcher.

### Inclusion/exclusion criteria of literature

Studies were eligible if they met the following inclusion criteria:1. All subjects of the study were adults (age>18) with OSA who were polysomnographically diagnosed on the basis of an apnea hypopnea index (AHI) equal to or greater than 5 events/h; 2. The intervention was an application of CPAP; 3. The study must report mean and SD (or SE) of adiponectin before and after CPAP. When multiple studies reported outcomes using the same patient group, the study with the largest population was included. If a study reported adiponectin values for more than one time point, we used the last available time point.

Abstracts, reviews, case reports, editorials, animal studies, conference articles, and non-English studies were excluded. If important data were ambiguous or lacked, the corresponding author was contacted by email, after twice non-response, the study was ruled out. Any disagreement between the two reviewers was resolved by discussing with a third reviewer.

### Data Extraction

Data were extracted by two independent researchers. The following variables were extracted from each study: first author, publication year, country of the study, sample size, patient inclusion criteria, participant characteristics, study design, mean daily CPAP usage time, duration of CPAP therapy, body mass index (BMI) and serum adiponectin levels before and after CPAP treatment.

### Statistical analysis

Statistical analysis was performed using Stata Version 12.0 (Stata Corporation, College Station, Texas, USA). Standardized mean difference (SMD) was used for analyzing the summary estimates. Q and I^2^ statistics were used to determine statistical heterogeneity among individual studies. Significant heterogeneity was indicated by p<0.10 or I^2^>50%. Random-effect (IV heterogeneity) model was performed to combine effect size if significant heterogeneity was observed; otherwise, the inverse variance-based fix-effect model was conducted[10]. Furthermore, to explore the possible sources of heterogeneity in CPAP treatment effects, subgroup analysis and the “leave one out” sensitive analysis of Patsopoulos et al[11] were conducted. Publication bias was presented using funnel plot and tested by “Begg test” and “Egger test”. A p<0.05 was adopted as statistical significance.

## Results

### Searching results

**Fig 1:**
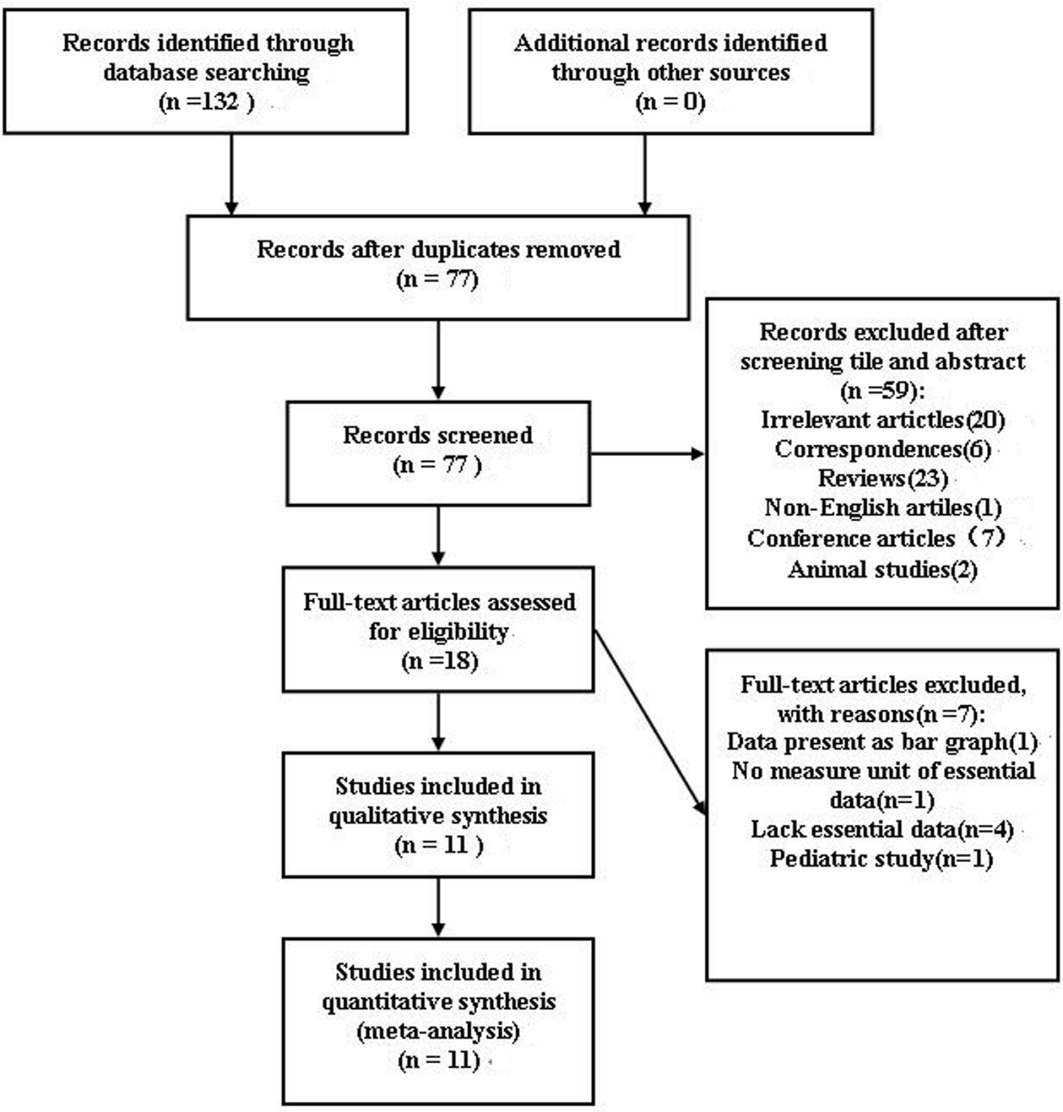
Flow diagram of study selection.

A total of 77 studies were retrieved to screen after searching duplication. After review of the titles and abstracts, 59 studies were excluded whereas 18 were considered to be potentially relevant. Of the 18 studies, 7 were excluded from the sample for the following reasons: 1 was pediatric study[12], 2 lacked of before and after CPAP adiponectin values[13, 14], 2 did not report data as mean and SD (or SE) [15, 16], the data of one study presented as bar graph[17], and one study had no measure unit of essential data[18]. The detailed steps of the literature search were shown in Fig 1.

### Characteristics of the included studies

Eleven studies (12 cohorts) involving 240 patients were included in this meta-analysis. One of them was randomized clinical trial (RCT)[19], the remaining studies were observational[20–29]. One study[27] reported results separately for obese group and non-obese group. The information of author, year of publication, nation, sample size, inclusion criteria, mean daily CPAP usage time, therapy duration and study design of each study was presented in Table 1. The information of mean age, AHI, lowestO_2_ saturation (LowSO_2_), BMI and adiponectin of each study was summarized in Table 2.

**Table 1:** Characteristics of include studies.

Study	Year	Nation	Sample size/male	Inclusion criteria	ventilation duration/night (h)	Therapy duration	Study design
Masserini	2006	Italy	13/7	AHI>10	NR	1d	Observational study
Sumi	2006	Japan	19/NR	AHI>20	NR	3-4d	Observational study
Zhang	2007	China	23/23	AHI>15	6–8	14d	Observational study
Takahashi	2008	Japan	27/25	AHI>20	>4	1M	Observational study
Vgontzas	2008	USA	16/16	AHI>20	4.6 ± 0.4	3M	Observational study
Carneiro	2009	Brazil	7/7	AHI>30	6.6±1.1	3M	Observational study
Garcia	2011	USA	20/17	AHI≥15	5.3±1.6	6M	Observational study
Sanchez-de-la-Torre(Obese)	2012	Spain	28/28	AHI≥20	5.7±1.4	3M	Observational study
Sanchez-de-la-Torre(Non-obese	2012	Spain	21/21	AHI≥20	5.7±1.4	3M	Observational study
Salord)	2013	Spain	21/NR	AHI>15	6	3M	Observational study
Kritikou	2014	USA	35/NR	AHI>10 (female) AHI>15 (male)	6.07±1.21	2M	RCT(cross-over)
Yoshikawa	2014	Japan	10/10	AHI≥5	Good compliance	3M	Observational study

Abbreviation: RCT = randomized controlled trial, AHI = apnea-hypopnea index, NR = not reported, d = day, M = month.

**Table 2:** Patients’ characteristics of the trials included in the meta-analysis.

Study	Age	AHI	LowSO_2_	Pre-BMI	Post-BMI	Pre-CPAP adiponectin	Post-CPAP adiponectin
Masserini	53.5±13.5	NR	NR	40.3±5.1	40.3±5.1	7.4±2.6ug/ml	6.7±2.8ug/ml
Sumi	NR	NR	NR	NR	NR	5.78±4.78mg/l	5.33±4.18mg/l
Zhang	49.5±12.4	33.8±11.4	72.8±8.1	26.8±2.5	26.7±2.3	3.95±0.99ug/ml	4.46±2.21ug/ml
Takahashi	48.3±10.7	48.2±14.8	65.2±16.5	30.1±4.6	NR(p = 0.26)*	3.55±1.37ug/ml	3.79±1.14ug/ml
Vgontzas	48.1±5.6	53.3±28.0	72.4±8.4	37.5±4.7	37.5±4.9	9.0±6.0ug/ml	10.4±13.6ug/ml
Carneiro	NR	91.0±25.7	71.2±5.3	46.1±7.4	46.8±6.9	7.1±4.5ng/ml	16.0±11.4ng/ml
Garcia	59.7±8.9	50.0±26.8	77.0±13.4	36.5±8.5	37.1±8.5	8.3±5.4ng/ml	8.2±5.4ng/ml
Sanchez-de-la-Torre(obese)	46.61±11.03	48.92±17.52	NR	34.34±3.49	NR(p>0.05)*	24.83±18.13μg/ml(28)	18.3±11.6μg/ml(22)
Sanchez-de-la-Torre(non-obese)	49.33±10.71	41.45±18.3	NR	25.02±1.22	NR(p>0.05)*	36.94±21.42μg/ml(21)	24.51±9.00μg/ml(16)
Salord	59(47–65) #	74.7(62–100)#	53.0(47.8–66)#	43.4±7.5(27)	42.0±6.4(27)	94.1±55ng/L	93.4±53ng/L
Kritikou	NR	38.49±21.65	82.11±6.57	28.55±0.57	29.02±0.57	7.32±0.63ng/mL	6.71±0.63ng/mL
Yoshikawa	NR	63.7 ± 14.9	NR	31.3 ± 4.5	NR(p>0.05)*	4.5±2.3ug/ml	6.8±2.1ug/ml

Abbreviation: AHI = apnea-hypopnea index, LowSO_2_ = lowest O_2_ saturation, BMI = body mass index, CPAP = continuous positive airway pressure, NR = not reported.

# presented as median (interqurtile range).

*p value extracted from original study.

### Pool analysis

**Fig 2:**
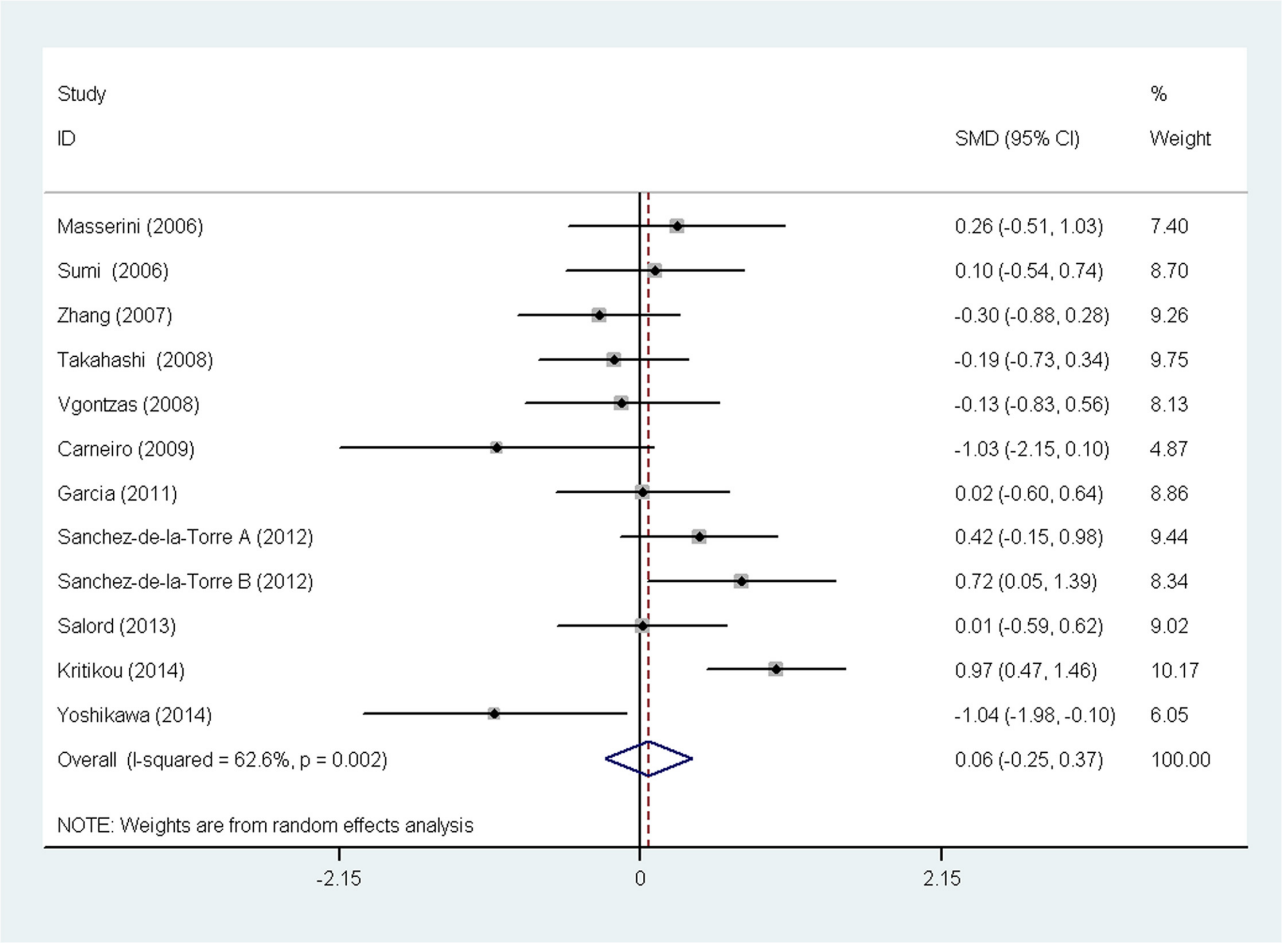
Comparison of adiponectin before and after continuous positive airway pressure therapy in the 11 included studies. Calculations based on a random-effect model. Abbreviation: SMD = standardized mean difference.

**Fig 3:**
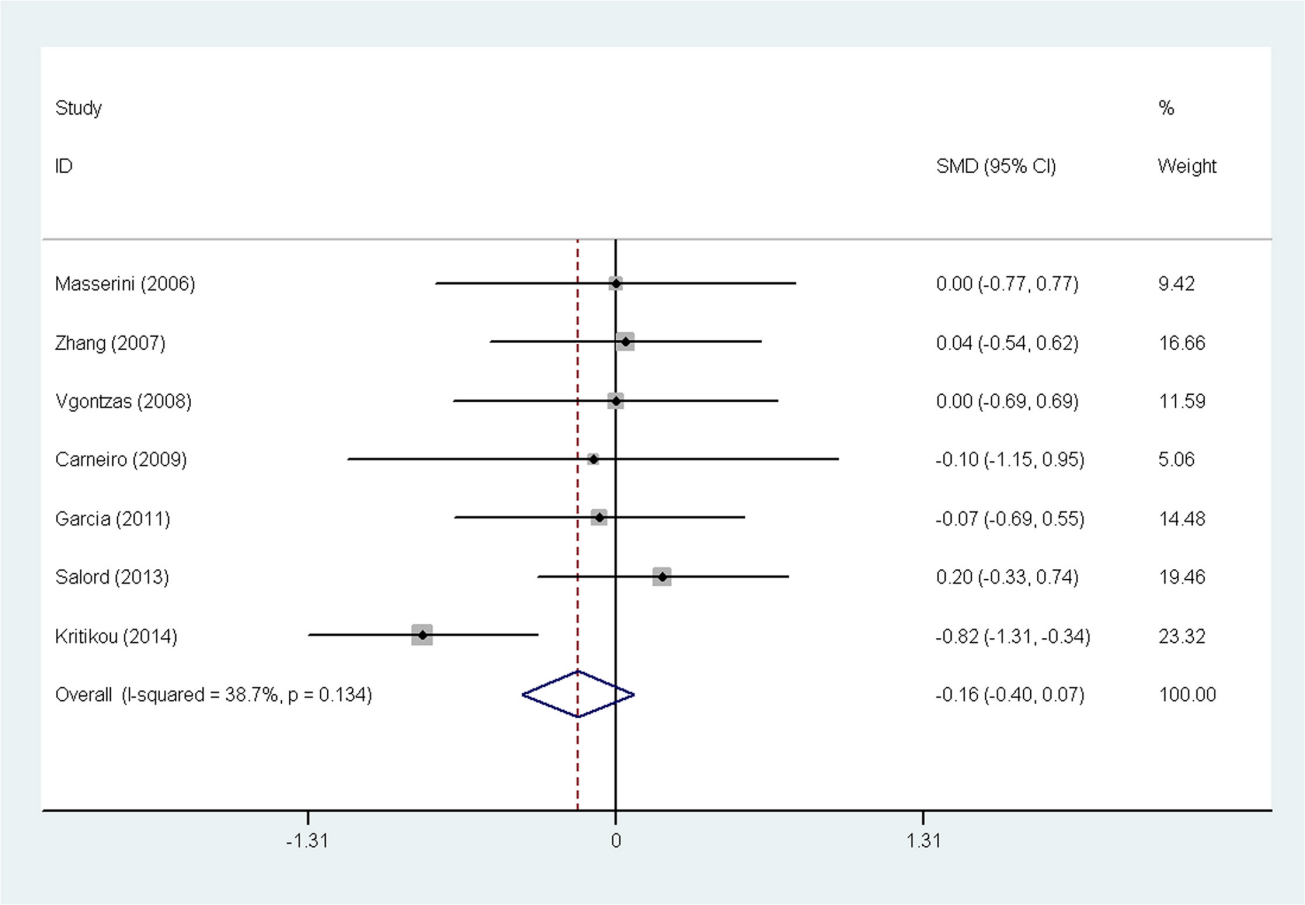
Comparison of BMI before and after continuous positive airway pressure therapy in the 7 included studies. Calculations based on a fixed-effect model. Abbreviation: SMD = standardized mean difference, BMI = body mass Index.

The heterogeneity test showed that there were significant differences across individual studies (chi-squared = 29.40, p = 0.002; I^2^ = 62.6%). Therefore, a random-effect model was used to combine effect size. No significant difference in adiponectin in OSA patients was observed before and after CPAP treatment after pooling the data with meta-analysis (SMD = 0.059, 95% confidence interval (CI) = −0.250 to 0.368, z = 0.37, p = 0.710) (Fig 2). A similar result (SMD = 0.129, 95% CI = −0.055 to 0.314, z = 1.37, p = 0.170) was also obtained from a fixed-effect model. A special meta-analysis regarding the impact of CPAP on BMI in patients with OSA was also performed. The result suggested that CPAP therapy had no significant impact on BMI in OSA patients (SMD = −0.161, 95% CI = −0.397 to 0.074, z = 1.34, p = 0.180) (Fig 3).

### Sensitivity and subgroup analyses

Sensitivity analysis showed that omitting any one of the studies at a time did not influence the overall result of the pooled analysis (data not shown). Considering the effectiveness of CPAP could be influenced by many factors, subgroup analyses were performed to access the impact of age (<50 and ≥50), baseline BMI (<35 and ≥35), severity of OSA (AHI≤50 and >50), CPAP therapy duration (<3 month and ≥3 month), sample size (<20 and ≥30) and racial differences (Asia and no-Asia). The results demonstrated that differences in age, BMI, AHI, CPAP therapy duration, sample size and racial differences did not affect CPAP efficacy (Table 3).

**Table 3:** The results of subgroup analyses.

Subgroup	Number of studies/patients	Heterogeneity			SMD			
		X^2^	P	I^2^(%)	SMD	95%CI	Z	P
Age								
<50	5/115	7.85	0.097	49.0	0.091	-0.288to0.469	0.47	0.639
≥50	3/54	0.29	0.865	0.0	0.074	-0.304to0.451	0.38	0.701
BMI								
<35	6/144	23.58	0.000	78.8	0.145	-0.393to0.684	0.53	0.597
≥35	5/77	3.65	0.455	0.0	-0.058	-0.376to0.260	0.36	0.722
AHI								
<50	5/134	15.88	0.003	74.8	0.323	-0.180to0.827	1.26	0.208
≥50	5/74	6.12	0.191	34.6	-0.290	-0.707to0.127	1.36	0.173
Follow time (M)								
<3	5/117	14.19	0.007	71.8	0.175	-0.325to0.675	0.69	0.492
≥3	7/123	14.48	0.025	58.6	-0.039	-0.457to 0.378	0.18	0.853
Sample size								
<20	5/65	7.42	0.115	46.1	-0.263	-0.754to0.227	1.05	0.292
≥20	7/175	17.43	0.008	65.6	0.239	-0.133to0.611	1.26	0.208
Race								
Asia	4/79	3.98	0.264	24.6	-0.262	-0.632to0.107	1.39	0.164
No-Asia	8/161	17.11	0.017	59.1	0.245	-0.120to0.611	1.31	0.189

Abbreviation: SMD = standardized mean difference, BMI = body mass index, AHI = apnea-hypopnea index, M = month.

### Publication bias

**Fig 4:**
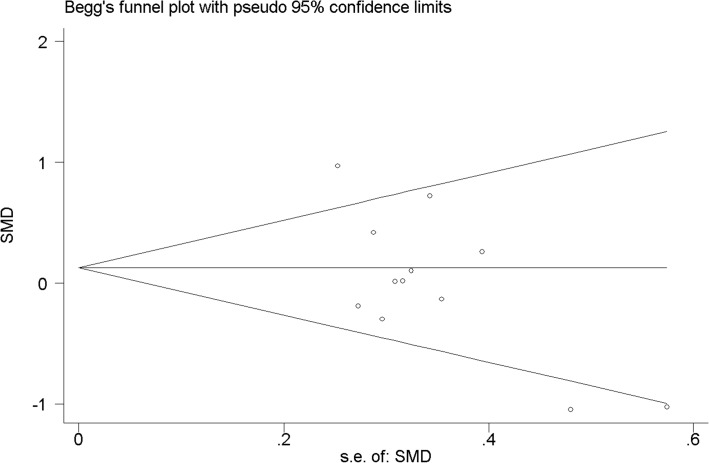
Funnel plots for assessing publication bias of studies included. Abbreviation: SE = standard error, SMD = standardized mean difference.

The funnel plot (Fig 4) showed that small publication bias seemed to exist. The Egger's test (p = 0.038) suggested evidence of publication bias, while the Begg's tests (p = 0.304) showed no evidence to support publication bias in the present meta-analysis. This difference in the results obtained from the two methods may be due to a greater statistical power of the Egger test[30]. In addition, trim-and-fill method showed that no study was needed to be correct for funnel plot asymmetry.

## Discussion

The present meta-analysis quantitatively evaluated the efficacy of CPAP on adiponectin in patients with OSA. The results of this meta-analysis of 11 studies showed that CPAP had no impact on adiponectin in OSA patients, independent of the change in body weight. Subgroup analysis did not find discrepant effect of CPAP therapy on adiponectin in OSA patients.

Adiponectin is an adipokine abundantly produced and secreted by adipose tissues and widely recognized for its anti-inflammatory, antiatherogenic, and cardioprotective effects[31]. In contrast to other adipocytokines, which are markedly upregulated in obesity, the serum levels of adiponectin are reduced in obese subjects. It has been suggested that low serum adiponectin levels were tightly associated with increased risk of atherosclerosis and myocardial infarction[32, 33]. Previous data from observational studies indicated that OSA was independently associated with adiponectin levels[6–8]. A cross-sectional analysis of 86 adult male patients suspected for OSA showed that serum adiponectin levels were significantly lower in the OSA group than in the control. On regression analysis, adiponectin was independently associated with OSA after controlling for BMI and other confounding factors[8]. In another study which matched body weight and age in 46 obese subjects grouped by AHI, adiponectin showed a trend to decrease according to the severity of OSA[6]. Consistently, experimental evidence from animal studies supported a mechanistic role of hypoxemia in reduction of adiponectin. In mice and cultured 3T3-L1 adipocytes, Nakagawa et al[18] found that exposure to hypoxia decreased adiponectin concentrations by inhibiting adiponectin regulatory mechanisms at both secretion and transcriptional levels. Consequently, reduced adiponectin levels associated with hypoxic stress may explain, in part, the development of cardiovascular disease in patients with OSA.

CPAP is considered to be the primary treatment for OSA[34]. As a noninvasive treatment of OSA, CPAP therapy could significantly reverse OSA associated hypoxia, sleep fragmentation, sympathetic activation and all these factors contribute to reduced adiponectin in OSA patients[18, 35, 36]. Thus, it is academically rational that CPAP therapy, to some extent, could increase adiponectin levels. However, the present meta-analysis suggested that CPAP treatment appeared to have no impact on adiponectin in OSA patients. The lack of change in adiponectin can be explained by the overwhelming influence of body mass on adiponectin secretion, which was unchanged during CPAP treatment. Another hypothesis to explain this negative result is the short therapy period. The treatment period of most of included studies did not exceed 3 months. A treatment period of 3 months was probably too short to have significant modification in adiponectin levels. Our subgroup analyses indicated that both CPAP therapy duration with<3 months and ≥3 months had no effect on this negative results. However, the effect of a longer duration of CPAP therapy on adiponectin remains to be explored.

The present analysis had several limitations that warrant additional comment. First, the number and size of studies included in this analysis was relatively small and larger and more studies would allow for more precise effect size estimation. Second, considerable heterogeneity was present among individual studies, but no exact source of heterogeneity was found. Third, most of the included studies were self control, only one RCT, pre-and post-treatment data rather than treatment and control groups data were extracted. It may, to some extent, weaken the impact of the work. Fourth, in our meta-analysis, different studies utilized a variety of measurement techniques for adiponectin. Considering adiponectin measured and reported differently, SMD was used for analyzing the summary estimates instead of the absolute levels of adiponectin. Finally, only papers published in English were enrolled, it may cause potential publication bias.

In summary, our meta-analysis did not demonstrate a significant effect of CPAP treatment in improving the adiponectin levels in OSA patients, without significant changes in BMI. Further prospective large-scale multicentre studies are needed to explore the longer treatment impact of CPAP therapy on adiponectin.

## Supporting Information

S1 PRISMA Checklist (DOC)

S1 PRISMA Flow Diagram (DOC)
