# Supplementary material for: Effect of Continuous Positive Airway Pressure on Adiponectin in Patients with Obstructive Sleep Apnea: A Meta-Analysis
Source: PLoS One. 2015 Sep 14;10(9):e0136837. doi: 10.1371/journal.pone.0136837 (PMC4569056; doi:10.1371/journal.pone.0136837)
Supplement: S1 PRISMA Flow Diagram — (DOC) [file pone.0136837.s002.doc]

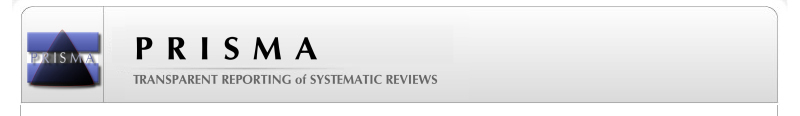
**PRISMA 2009 Flow Diagram**

**Screening**

**Included**

**Eligibility**

**Identification**

**Records identified through database searching
(n =132 )**

**Additional records identified through other sources
(n = 0)**

**Records after duplicates removed
(n = 77)**

**Records screened
(n = 77 )**

**Full-text articles assessed for eligibility
(n =18)**

**Full-text articles excluded, with reasons(n =7):**

**Data present as bar graph(1)**

**No measure unit of essential data(n=1)**

**Lack essential data(n=4)**

**Pediatric study(n=1)**

**Studies included in qualitative synthesis
(n = 11 )**

**Studies included in quantitative synthesis (meta-analysis)
(n = 11)**

**Records excluded after screening tile and abstract
(n =59):**

**Irrelevant artictles(20)**

**Correspondences(6)**

**Reviews(23)**

**Non-English artiles(1)**

**Conference articles（7）**

**Animal studies(2)**
